# Chronological Analysis of Visual Field and Optic Nerve Thickness for the Early Diagnosis of Bilateral Chronic Open-Angle Glaucoma With a Masquerading Growing Pituitary Tumor

**DOI:** 10.7759/cureus.112741

**Published:** 2026-07-15

**Authors:** Emily Kang, Ming Chen

**Affiliations:** 1 Department of Medicine, University of Hawaiʻi John A. Burns School of Medicine, Honolulu, USA

**Keywords:** bitemporal visual field defect, compressive optic neuropathy, glaucoma, optical coherence tomography (oct), pituitary tumor

## Abstract

This case illustrates that a pituitary tumor can be masked by coexisting glaucoma, and that early differential diagnosis using optical coherence tomography (OCT), Central 24-2 threshold visual field testing, and magnetic resonance imaging (MRI) to facilitate timely surgical intervention might avoid irreversible vision loss.

A 73-year-old female was initially diagnosed with cataracts and glaucoma and monitored by retinal nerve fiber layer (RNFL) measurements using OCT, with readings of 97 μm in the right eye and 85 μm in the left eye in 2010. This case was followed continuously by the author for over 17 years. Although her intraocular pressure remained well controlled, her left eye RNFL progressively declined, dropping to 72 μm in 2022 and further to 62 μm in 2025. Visual field testing in 2016 revealed bitemporal defects, including a glaucomatous arcuate defect, which became increasingly pronounced by 2020 and, by 2025, demonstrated severe defects in the left eye, inconsistent with controlled glaucoma. MRI in 2025 confirmed a 2.2 cm adenoma compressing the optic chiasma with left optic nerve atrophy.

In retrospect, review of the chart revealed early warning signs of a brain lesion, and the tumor was unfortunately identified only after significant vision loss had already occurred, underscoring that early diagnosis and timely surgery can prevent and sometimes reverse vision loss. Any atypical glaucoma presentation or inexplicable change in a patient with controlled glaucoma should therefore be carefully evaluated to exclude pituitary or other intracranial tumors, particularly through diligent visual field review and optic nerve examination.

Glaucoma is fundamentally an optic nerve disease, and multidisciplinary cooperation is essential: neurologists managing pituitary tumor patients should refer them to ophthalmologists to rule out coexisting glaucoma, while ophthalmologists encountering unusual glaucoma cases should refer patients to primary care physicians or neurologists to exclude intracranial pathology. This report aims to remind clinicians of this rare but well-documented condition and highlight early diagnostic clues to prevent the pituitary tumor from being masked by a narrow specialty focus.

## Introduction

Glaucoma is an optic nerve disease characterized by progressive cupping and visual field loss, often linked with intraocular pressure (IOP) elevation. However, there are many other optic nerve diseases that can mimic glaucoma. Besides high IOP causing optic neuropathy, congenital optic disc anomalies, hereditary optic neuropathy, and compressive lesions can mimic glaucoma with or without high IOP [1-4]. Among the above illnesses, compressive optic neuropathy is the most difficult to diagnose, as an MRI can be expensive and hard to obtain.

A pituitary tumor, a benign and slowly growing tumor right on the optic chiasm, can coexist with glaucoma. In addition to visual changes, pituitary adenomas may present with headaches or hormonal disturbances, though these may be subtle or absent depending on tumor type and patient demographics [5]. According to a study [5], the incidence of pituitary tumors is about three cases per 100,000. A Hawaii-based study showed that pituitary tumors are more common in Native Hawaiian and Pacific Islander individuals than in White individuals, with an overall 6.8% of a cohort of 323 cases of all brain tumors being a pituitary adenoma [6]. The exact incidence of pituitary adenoma in Hawaii remains unknown.

Both pituitary tumors and glaucoma can be symptom-free until the late stage, when vision and visual field are jeopardized. Besides controlling IOP, early resection of the pituitary tumor may prevent loss of vision.

## Case presentation

Initial presentation and baseline glaucoma management (2010-2017)

**Figure 1:**
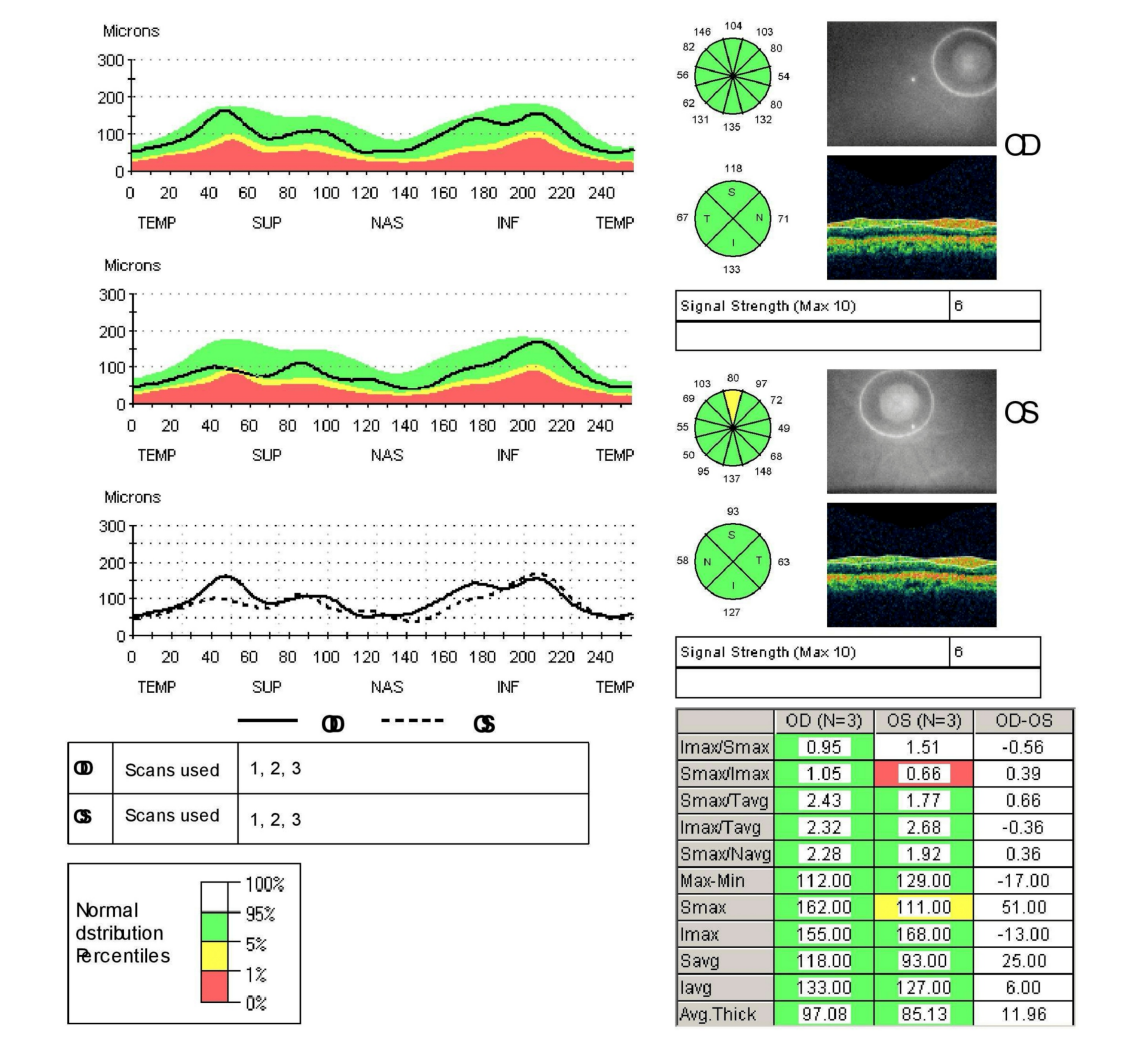
Optical coherence tomography from 2010. Initial retinal nerve fiber layer (RNFL) thickness was 97 μm in the right eye (OD) and 85 μm in the left eye (OS).

A 73-year-old female has been continuously followed by the author in the State of Hawaii for 17 years since her immigration. Her chief complaint was blurred vision. The patient’s oral history from 2010 revealed a possible brain tumor, but the MRI was normal, and the patient was not treated or followed up. At that time, her retinal nerve fiber layer (RNFL) thickness was 97 μm in the right eye and 85 μm in the left eye (Figure 1). Her grade of cataract in 2010 was 2+ cortical in the left eye, with visual acuity of 20/30, refraction of +1.25 sphere, +1.00 cylinder at axis 1°, IOP of 15 mmHg, and cup-to-disc ratio (CDR) of 0.50. The patient received cataract surgery in 2010 on the left eye due to a continuous complaint of blurred vision, and the gonioscope showed a narrow component with a mostly open angle.

**Figure 2:**
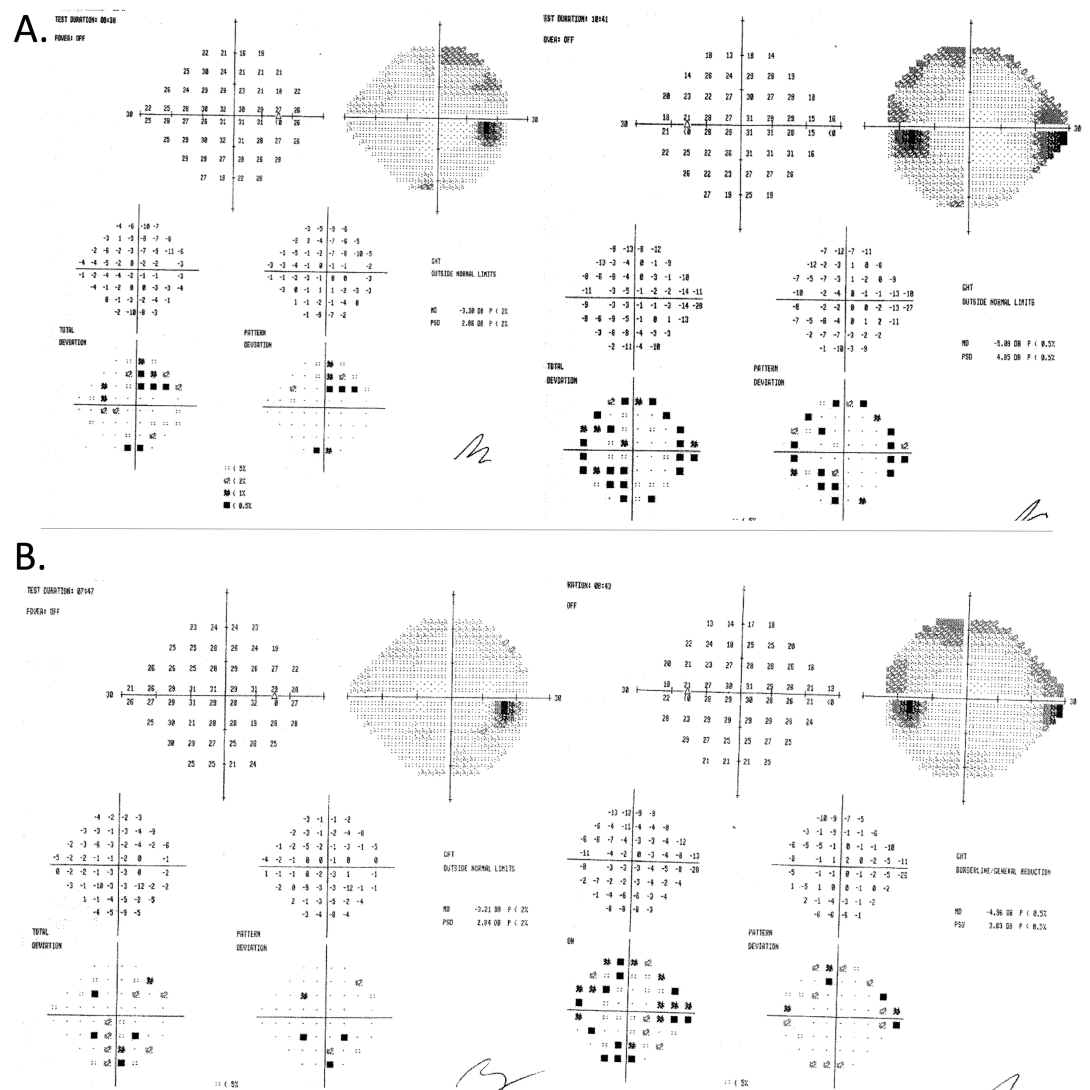
Humphrey visual field (HVF) test from 2016 and 2017. Central 24-2 threshold HVF tests from (A) June 2016 and (B) July 2017 demonstrated some bitemporal component with arcuate defects consistent with the initial diagnosis of chronic open-angle glaucoma. The left eye also showed nasal step and an enlarged blind spot.

Records from 2015 revealed that her IOP was 19 mmHg in the left eye and 21 mmHg in the right eye, and RNFL was 99 μm in the right eye and 83 μm in the left eye. Corneal hysteresis (CH) value of 7.5 mmHg and CDR of 0.69 were recorded for her left eye. Based on these findings and Central 24-2 threshold Humphrey visual field (HVF) test results from 2016 and 2017, the patient was diagnosed with chronic open-angle glaucoma of the left eye (Figure 2). Subsequent treatments included Xalatan eye drops, selective laser trabeculoplasty (SLT), and a Xen implant, with improved results in the left eye with an IOP of 14.4 mmHg, CH of 10.0 mmHg, and CDR of 0.71 in 2018.

Disease progression despite intraocular pressure control (2020-2023)

**Figure 3:**
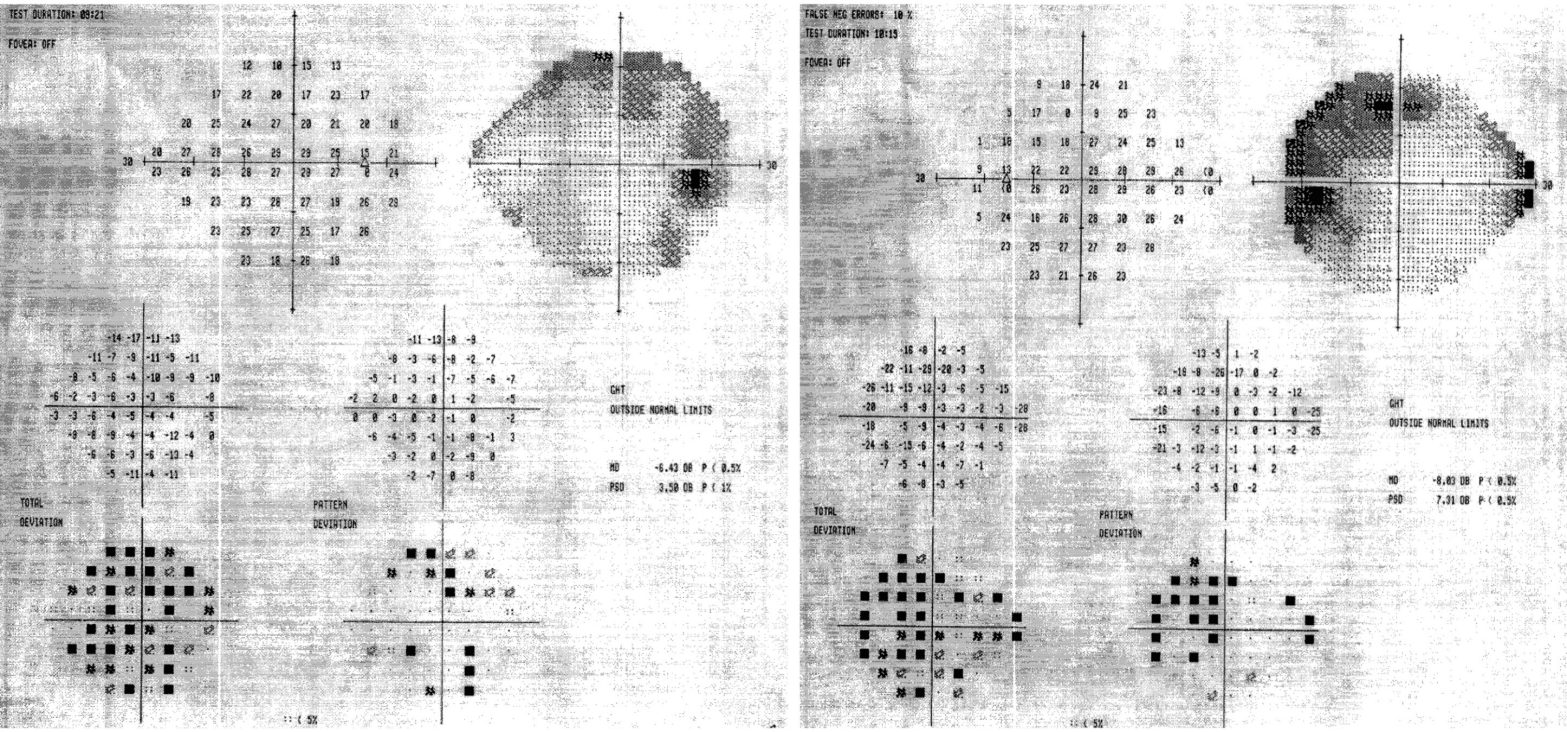
Humphrey visual field (HVF) test from 2020. Central 24-2 threshold HVF from May 2020 revealed an emerging bitemporal pattern, though still masked by pronounced arcuate defects.

In 2020, the Central 24-2 threshold visual field showed a bitemporal defect, though it remained partially masked by pronounced arcuate defects (Figure 3). The IOP in 2020 remained controlled at 14.1 mmHg in the left eye, with a CH value of 9.7 mmHg and CDR of 0.72.

**Figure 4:**
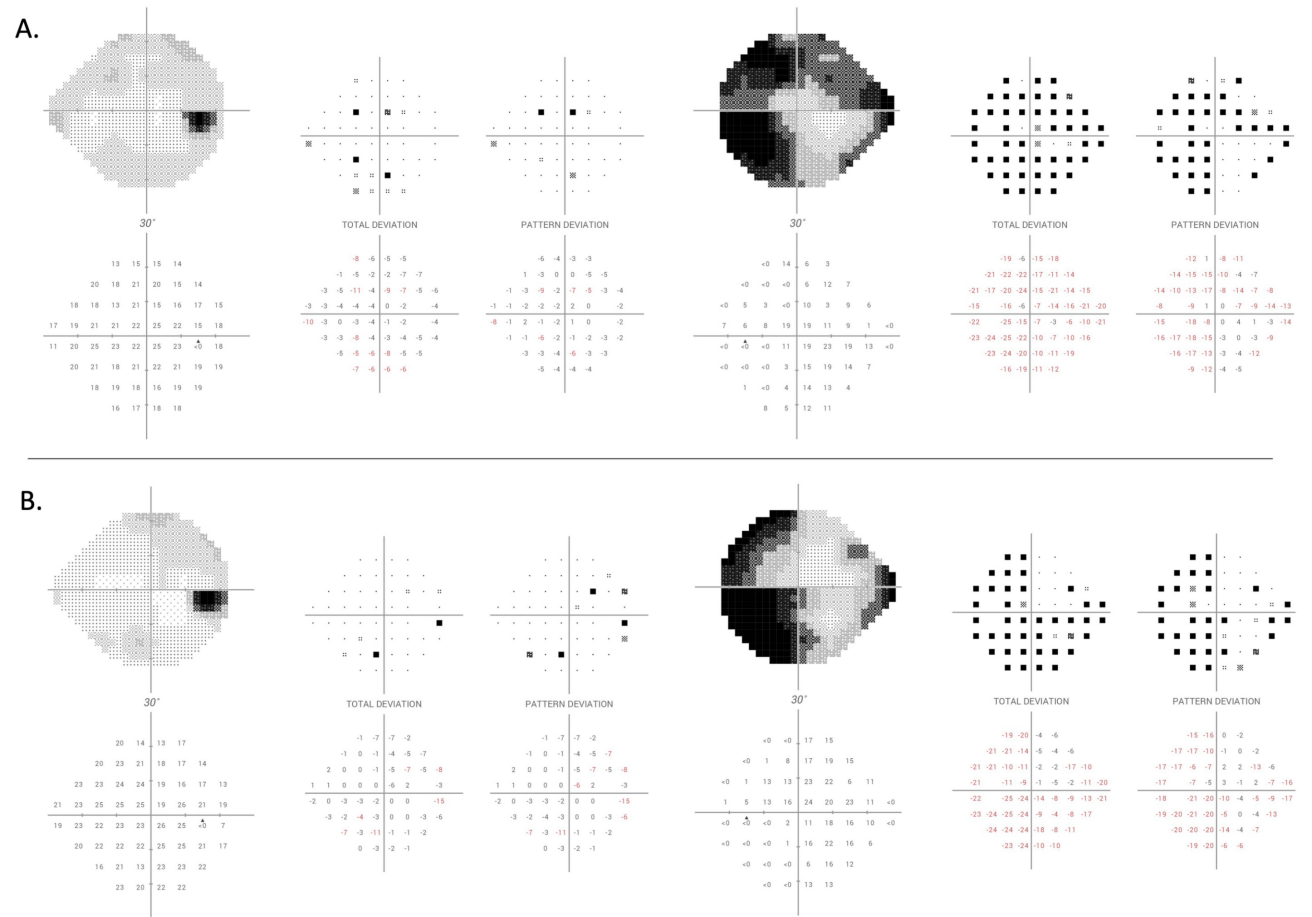
Longitudinal Humphrey visual field (HVF) test from 2023. Longitudinal HVF tests from (A) January 2023 and (B) December 2023 revealed bitemporal component defects, more evident on the left side, rather than a typical bitemporal defect.

**Figure 5:**
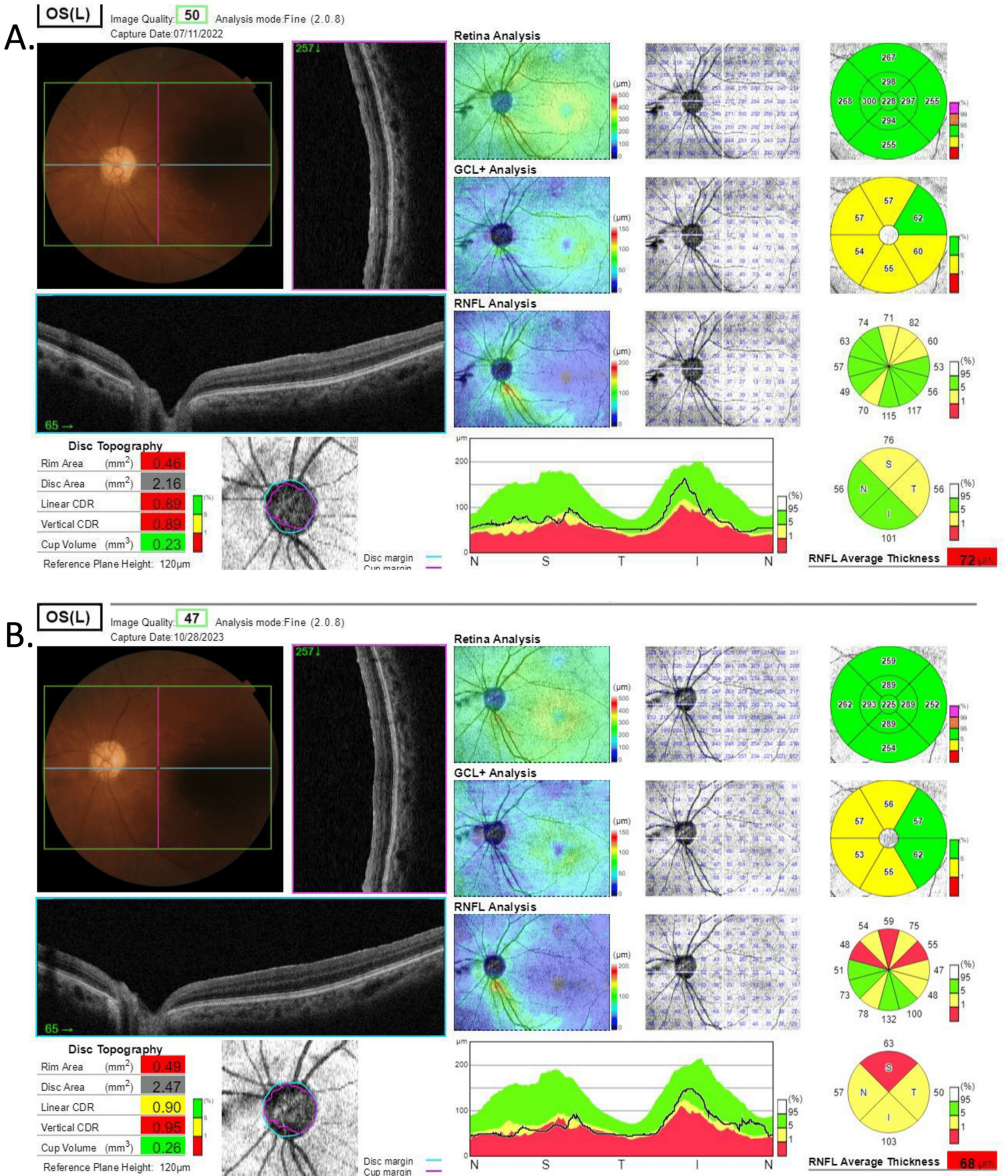
Optical coherence tomography (OCT) from 2022 and 2023. (A) OCT from July 2022 showed a significant decline in retinal nerve fiber layer (RNFL) thickness to 72 μm in the left eye (OS). (B) OCT from October 2023 showed a steady decline to 68 μm in the left eye (OS) in the RNFL.

Bitemporal component defects continued to develop in 2023 (Figure 4). Although the IOP in 2023 remained controlled at 13.4 mmHg in the left eye, the CH value decreased to 8.0 mmHg, and the CDR value increased to 0.94. The RNFL thickness in the left eye decreased to 72 μm by 2022 (Figure 5A) and further to 68 μm by 2023 (Figure 5B). The pituitary tumor was still not under suspicion by the clinician at this time.

Neuro-ophthalmic correlation and pituitary adenoma diagnosis (2024-2025)

**Figure 6:**
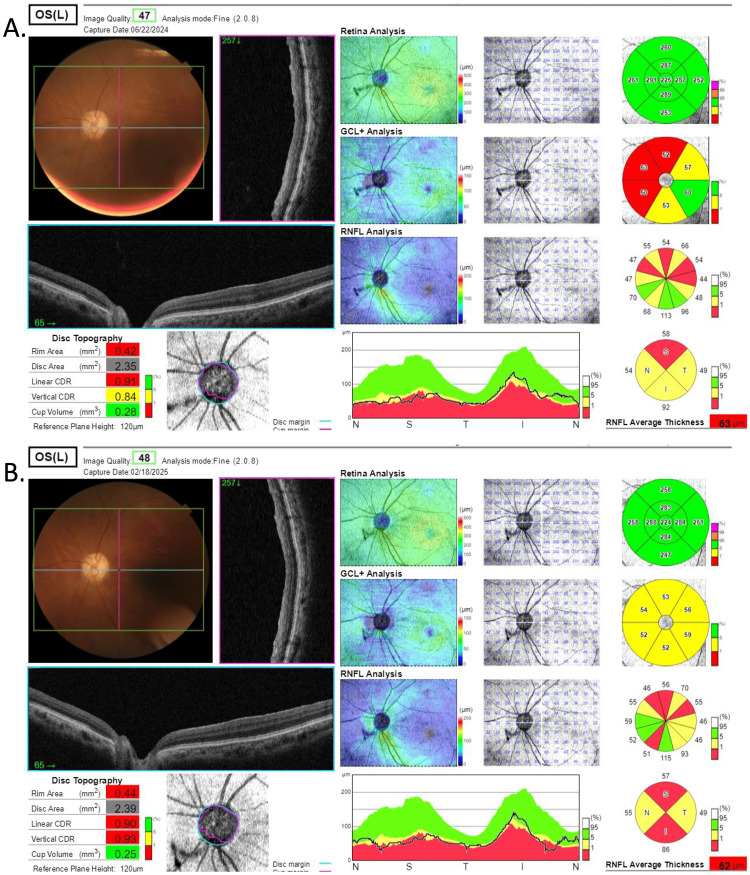
Optical coherence tomography (OCT) from June 2024 and February 2025. (A) OCT from June 2024 showed the steady decline to 63 μm in the left eye (OS). (B) OCT from February 2025 showed advanced left optic nerve atrophy down to 62 μm in the left eye (OS).

**Figure 7:**
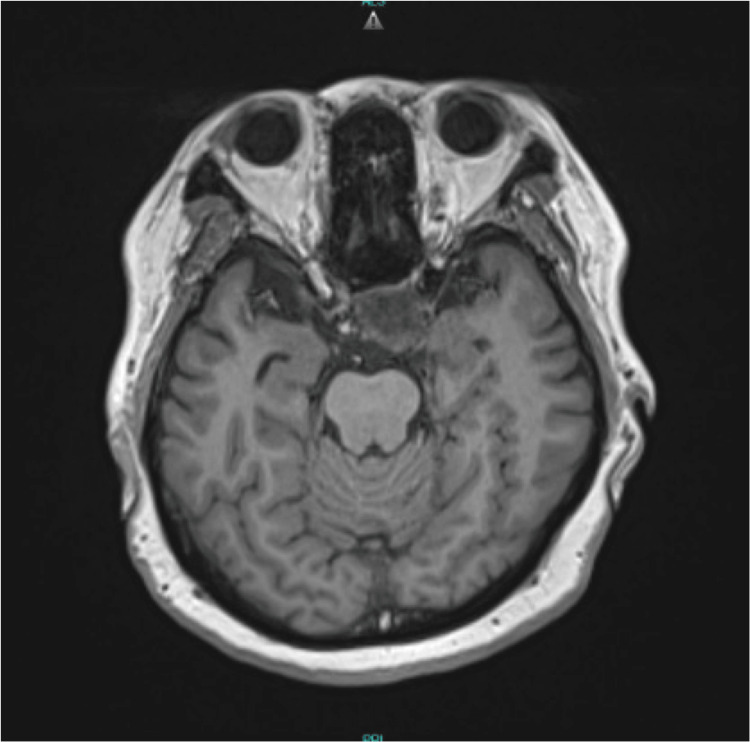
Brain magnetic resonance imaging (MRI) from February 2025. MRI revealed a 2.2 cm pituitary adenoma with significant compression of the optic chiasm.

Left eye RNFL continued to thin to 63 μm in 2024 (Figure 6A). It was not until February 2025 that an eye exam revealed a pale disc with a Marcus Gunn pupil (relative afferent pupillary defect) in the left eye, prompting a brain MRI. MRI confirmed a 2.2 cm adenoma compressing the optic chiasm with left optic nerve atrophy (Figure 7). The vision was 20/40 in the right eye and 20/100 in the left eye. OCT imaging documented advanced left optic nerve atrophy, with RNFL reaching 62 μm in the left eye (Figure 6B), correlating with the clinical finding of a 2.2 cm pituitary adenoma. 

Final clinical status

**Figure 8:**
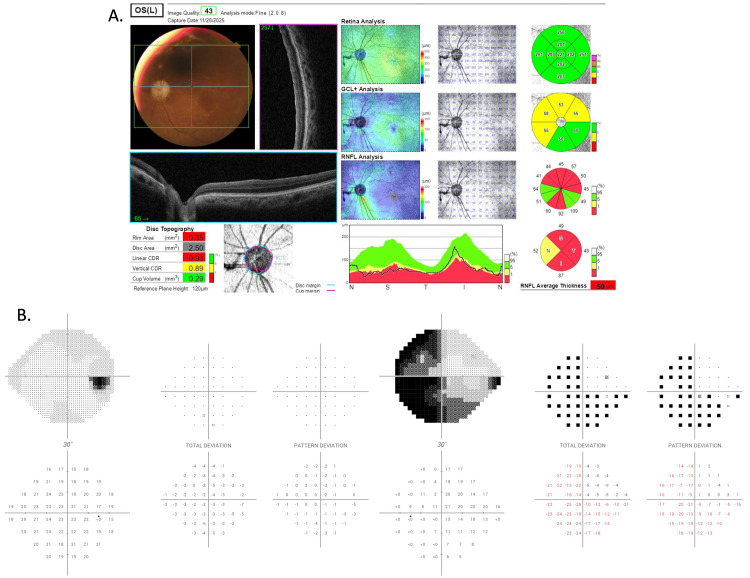
Optical coherence tomography (OCT) and Humphrey visual field (HVF) test from 2025. (A) OCT in November 2025 showed the terminal thinning of the retinal nerve fiber layer (RNFL) to 59 μm in the left eye (OS). (B) Humphrey visual field (HVF) test in August 2025 showed a severe vertical defect in the left eye (OS), with a bilateral arcuate defect.

Following the diagnosis of pituitary adenoma, the patient was referred to a neurosurgeon for surgery, but unfortunately, she declined the surgical procedure recommended by the neurosurgeon. Long-term comparison between 2010 and 2025 reveals a total unilateral decrease in the left eye RNFL from 85 μm to 59 μm (Figure 8A), with final visual field testing showing a dense bitemporal/arcuate combination vertical defect (Figure 8B) more evident in the left eye. The case concludes with permanent structural and functional deficits due to the prolonged compression and the patient's decision to forgo surgery.

## Discussion

This case highlights the diagnostic challenges when compressive optic neuropathy coexists with glaucoma, demonstrating how pituitary adenomas can be overlooked despite progressive visual deterioration. A pituitary tumor mainly has two types: functioning, which presents with hormonal symptoms, and non-functioning, which shows minimal hormonal changes. Functioning tumors, such as prolactinomas, the most common functioning type, typically present with amenorrhea and galactorrhea, but these symptoms may be absent in postmenopausal patients [7], as was the case here, potentially masking an important diagnostic clue.

It is worth noting that pachymetry, IOP phasing, and color vision assessment were unavailable in the clinical setting where this patient was evaluated, which may have contributed to the delayed recognition of the underlying pituitary adenoma and the need for imaging. However, corneal hysteresis was measured and showed a low value, which may result in a misreading of IOP as lower. Careful review of patient history, family history, visual field patterns, and OCT findings can facilitate earlier diagnosis of compressive lesions in cases with high suspicion for atypical or inexplicable glaucoma, prompting timely brain MRI [1,8-10]. However, as a brain CT scan may not show the lesion, the expensive MRI scan becomes necessary, and it may be difficult to get insurance approval. Some of the criteria below may provide hints for early diagnosis, as learned from this case.

Clinical recognition of compressive optic neuropathy

There are several key clinical signs that can raise suspicion for compressive optic neuropathy in patients being treated for glaucoma. First, progressive, rapidly deteriorating glaucoma despite controlled IOP is one of the most significant warning signs [1,8,10]. In this case, the RNFL continued to thin from 85 μm to 62 μm over 15 years despite IOP control at 14 mmHg. Second, optic disc pallor that is disproportionately greater compared to the cupping is another key sign to look out for [1,3]. This pallor of disc finding was prominent in our case and served as an early clue for neuroimaging. Additionally, the presence of relative afferent pupillary defect (RAPD) or early color vision loss that is disproportionate to the visual field should prompt further investigation for non-glaucomatous causes [3] since symptoms are more characteristic of ischemic or compressive optic neuropathies.

Visual field and OCT pattern recognition

Patterns in visual field defects and OCT changes can provide crucial diagnostic clues when distinguishing glaucoma from compressive lesions. First, while arcuate visual field defects are characteristic of glaucoma, they can also present in compressive lesions. In addition, the presence of typical or atypical bitemporal visual field components with vertical meridian defects [1] or central and cecocentral scotomas [3] should raise concerns about non-glaucomatous pathologies, such as chiasmal compression. In this case, subtle bitemporal components [11] were present as early as 2016 but were not initially recognized as suspicious for compressive pathology, as they were masked by the glaucomatous defect that showed heavily on the nasal side. Careful scrutiny is required to distinguish negative numerical values in the bitemporal fields, which consistently show larger deficits than adjacent “normal” areas. This pattern, which can often be overlooked, explains why such compressive lesions are frequently missed, emphasizing the need for meticulous quantitative review of visual field data beyond qualitative pattern recognition. Another clinical pattern to look out for is when optic disc appearance and cupping do not correlate with the degree of visual field loss [1,9]. Lastly, the OCT pattern of horizontal RNFL thinning, particularly when asymmetric and progressing unilaterally, does not match the typical superior- and inferior-thinning pattern seen in glaucoma [1,2].

Indications for neuroimaging

The decision to pursue neuroimaging in glaucoma patients requires careful clinical judgment because MRI is costly and may require insurance authorization. Based on the findings of this case and the broader literature, neuroimaging should be considered if any of the following clinical patterns are present: (1) visual field defects that do not respect the vertical meridian, or other atypical defect patterns such as bitemporal components or central/cecocentral scotomas; (2) unexplained or disproportionate vision loss not accounted for by IOP or structural glaucomatous changes; (3) optic disc pallor exceeding excavation, where the neuroretina rim appears pale but is not deeply cupped; (4) decreased color vision disproportionate to the degree of visual field loss.

Studies have shown that approximately 7% of patients with atypical unilateral glaucoma will have abnormal findings on MRI [8,10]. This prompts a significant diagnostic yield in carefully selected cases, especially when multiple concerning features discussed above coexist. In cases when uncertainty persists despite evaluation, a referral to a neuro-ophthalmologist can provide guidance in distinguishing glaucomatous from non-glaucomatous optic neuropathy [9,11].

## Conclusions

This case report highlights the need for early detection of pituitary tumors masquerading as glaucoma through careful attention to patient history, visual field assessments, and OCT findings, with a high level of suspicion. A combination of unilateral RNFL deterioration, bitemporal visual field defects, and disease progression despite controlled IOP should prompt consideration of early neuroimaging. Clinicians should remain attentive to atypical clinical features with inexplicable changes and adopt a low threshold for brain MRI when multiple concerning patterns are present. The consequences of delayed diagnosis, including irreversible visual loss and diminished patient quality of life, significantly outweigh the challenges associated with obtaining timely neuroimaging.
